# Persons with dementia missing in the community: Is it wandering or something unique?

**DOI:** 10.1186/1471-2318-11-28

**Published:** 2011-06-05

**Authors:** Meredeth A Rowe, Sydney S Vandeveer, Catherine A Greenblum, Cassandra N List, Rachael M Fernandez, Natalie E Mixson, Hyo C Ahn

**Affiliations:** 1 College of Nursing, University of Florida, Gainesville, FL, USA; 2 James A. Haley Veterans Hospital, Tampa, FL, USA; 3 College of Nursing, University of South Florida, Tampa, FL, USA

## Abstract

**Background:**

At some point in the disease process many persons with dementia (PWD) will have a missing incident and be unable to safely return to their care setting. In previous research studies, researchers have begun to question whether this phenomenon should continue to be called wandering since the antecedents and characteristics of a missing incident are dissimilar to accepted definitions of wandering in dementia. The purpose of this study was to confirm previous findings regarding the antecedents and characteristics of missing incidents, understand the differences between those found dead and alive, and compare the characteristics of a missing incident to that of wandering.

**Methods:**

A retrospective design was used to analyse 325 newspaper reports of PWD missing in the community.

**Results:**

The primary antecedent to a missing incident, particularly in community-dwelling PWD, was becoming lost while conducting a normal and permitted activity alone in the community. The other common antecedent was a lapse in supervision with the expectation that the PWD would remain in a safe location but did not. Deaths most commonly occurred in unpopulated areas due to exposure and drowning. Those who died were found closer to the place last seen and took longer to find, but there were no significant differences in gender or age. The key characteristics of a missing incident were: unpredictable, non-repetitive, temporally appropriate but spatially-disordered, and while using multiple means of movement (walking, car, public transportation). Missing incidents occurred without the discernible pattern present in wandering such as lapping or pacing, repetitive and temporally-disordered.

**Conclusions:**

This research supports the mounting evidence that the concept of *wandering*, in its formal sense, and *missing incidents *are two distinct concepts. It will be important to further develop the concept of missing incidents by identifying the differences and similarities from wandering. This will allow a more targeted assessment and intervention strategy for each problem.

## Background

One of the most challenging issues related to the care of persons with dementia is the occurrence of leaving the caregiving environment without supervision. The term wandering is frequently used in the literature as a broad term encompassing a diverse set of behaviors including aimless locomotion with a repetitive pattern such as lapping or pacing, hyperactivity, and excessive walking, as well as leaving a safe environment and becoming lost alone in the community [1]. The risk of missing incidents is significant and the Alzheimer's Association estimates that up to 60 percent of PWD will 'wander' into the community at some point during the course of their disease [2,3].

As the population ages and the prevalence of illnesses causing dementia increases, communities will increasingly have to manage the problem of PWD missing in the community. Unable to find their way home or to a safe location, these individuals are then dependent upon a good Samaritan to identify their predicament and intervene, or to be found by law enforcement personnel searching for them. While most of these individuals will be found alive and unharmed, some of them will die from exposure, drown, or suffer injuries or fatalities after being involved in a car accident as either a driver or a pedestrian.

Communities have begun to respond with limited efforts to find lost PWD who left by car by enacting programs such as those that use community alerts to notify citizens of a missing individual (i.e., Silver Alert programs in the United States or A Child is Missing in Canada)[1]. It is crucial to augment these beginning efforts and enact programs that support all missing PWD with evidence-based strategies that promote a rapid discovery of the missing individual. Alzheimer's Association's literature indicates that up to half of lost PWDs who are not found within 24 hours suffer serious injury or death [2]. Community alert programs may reduce the time it takes to find a missing PWD and the serious consequences that occur as a result of exposure. Furthermore, evidence-based preventive strategies may also reduce the negative outcomes of a missing incident such as higher rates of institutional placement that are correlated with caregiver stress due to missing incidents [2].

The purpose of this study was to advance the early findings from previous research regarding antecedents of PWD missing in the community and characteristics of how missing PWD are found, and to compare in a single study the differences between PWD who were found alive and those who were found dead. Finally, we will examine the conceptual differences between the definition of wandering [3] and the characteristics of missing incidents.

The previous research in this area consists of studies that focus specifically on PWD lost in the community [2,4-8] as well as studies that focus specifically on wandering in formal care settings but include some information on exits or elopements [9-11]. In the review of this research, four factors were found to be characteristic of incidents in which a person with dementia who was missing.

First, while missing incidents are commonly called "wandering", PWD seem to go missing during usual activities, such as going for a routine walk or drive or being out in the community with the caregiver [12,13]. PWD also went missing when they were left alone in a familiar location temporarily during a usual activity, such as when left watching television while the caregiver was elsewhere in the home or when allowed to rest in a store or a parked car while the caregiver shopped. Occasionally, exits were directly related to the desire to go to a different location [14]. Other PWD went missing when they were intentionally left alone, either at home or while sleeping at night [5].

The second factor to emerge was the unpredictability and unexpectedness of the missing incident. In a prospective study, there were few predictors of which PWD would leave the home unattended and require an escort to return [2]. The fact that these incidents seemed to occur when the caregiver permitted an activity or knowingly left the PWD alone indicates that the incident was expected to end uneventfully and helps illuminate the unpredictable timing of the event [13]. Furthermore, while some individuals went missing on multiple occasions, the majority had a singular event. It is possible that subsequent missing incidents were prevented due to changes instituted after the initial event. Caregivers report fairly minor changes in routine after a missing incident however, such as informing neighbors and ensuring that the PWD had an identification bracelet, strategies unlikely to prevent a future incident [14].

Third, the missing incident always occurred in the absence of supervision, sometimes a lapse of just a few minutes. In general, lapses of supervision appear to have been planned by the caregiver and based on a previously successful outcome in that same type of situation.

Fourth, there were notable differences in circumstances between individuals who were found alive and those found dead. In studies that focused primarily on those found dead, the large majority were found in locations that were secluded and away from other people [4,7]. For instance, many of those who died while missing were found in woods and other natural areas such as ditches, fields, or abandoned structures. Those who died had often made additional attempts to seclude themselves or did not respond to (or hid from) searchers who were very close to them. This presents a significant contrast to those found alive, who were generally found in populated areas such as neighborhoods, streets, sidewalks, or businesses [13].

The evidence is beginning to accumulate that a missing incident and the phenomenon of wandering may be conceptually different. A team of researchers proposed the definition of wandering in 2007 as:

a syndrome of dementia-related locomotion behavior having a frequent, repetitive, temporally-disordered, and/or spatially-disordered nature that is manifested in lapping, random, and/or pacing patterns some of which are associated with eloping, eloping attempts, or getting lost unless accompanied" [3]p. 696.

There is no empiric evidence that wandering is directly associated with exiting or eloping. Although the overall body of research on wandering is both recent and small, there is some evidence that exits from home and formal care settings are purposeful events [10,14]. Rowe and Bennett have proposed that it is incorrect to use the terms wandering and becoming lost, or missing, interchangeably as not all PWD with dementia who wander will become lost and not all PWD who become lost exhibit wandering behaviors [9]. This is an important time to determine the differences between these concepts as the definitions are being developed and formalized.

A goal of this research was to determine how the characteristics of wandering match the characteristics of missing incidents. The aims of the study were to:

1. Extend previous research findings regarding the antecedents and characteristics of missing incidents.

2. Describe differences between PWD who were found alive and those who were found dead in a sample population.

3. Determine whether characteristics of a missing incident are similar to or different from an accepted definition of *wandering*.

## Methods

### Study Design and Sample

We used a retrospective descriptive design, conducted by gathering data from US newspaper articles published over 48 consecutive months from July 2003 through June 2008. The articles described incidents in which PWDs went missing and/or were found. To locate reports, we used three Internet search engines: NexusLexis Academic, Dow Jones Interactive, and Google. The largest number of articles were found with the search terms "Alzheimer's and (missing or lost)" or "dementia and (missing or lost)". Most of the articles came from local newspapers from the area in which the PWD either went missing or was located. Inclusion criteria for reports were: the person described had a diagnosis or suspected diagnosis of dementia and required a search effort to be found, and the report included information about when the individual went missing and when he or she was found, and contained valid data for at least 50% of the study variables. To collect the data, we retrieved the original newspaper articles. All cases that met inclusion criteria were used. A total of 325 cases were identified and used in the analysis. Individuals' names and hometown were collected to ensure that each case we analyzed was a unique event.

### Measurement and Data Analysis

We collected information on the following variables: gender, age, city, state and zip code of place last seen (PLS), time last seen, circumstances of situation in which the PWD was missing, mode of travel (car, walking, etc.), description of the place found, who found the individual, length of time missing, distance from PLS, types of search strategies used/description of how the individual was found, and cause of death in some cases. To understand the antecedent activity, we used both the wording of the event as well as categorical coding using a list of categories generated from a previous study (such as missing while on a routine activity, left home during night). To determine the total time missing, we used the number of days since often the exact time the PWD was missing was not known. For instance, actual times wouldn't be known if the individual left in the middle of the night, when left alone, or on a routine activity and wasn't determined to be missing until a time after he/she should have returned. Thus, time missing is reported as follows: 0 days = found same day as missing, 1 day = found the next day, etc.

For data analysis, we used descriptive statistics, X^2 ^and t-tests, computed using PASW 18.0 (SPSS Inc., Chicago, IL). The alpha was set at *p *= 0.05.

## Results

Demographic characteristics of the 325 cases are displayed in Table 1. The sample has a notably higher proportion of males (almost 2/3^rds^), particularly considering that females predominate the typical population of PWD (approximately a 2:1 female to male ratio) [15]. The type of dementia diagnosed was not able to be determined due to the data source. Nine incidents occurred in which a married couple was missing together. There was a large range of ages, from younger people with dementia to the very old, indicating that a missing incident can occur across all ages including the oldest old who can be quite frail. The data came from forty-six states with only Alabama, Delaware, Iowa, and Montana unrepresented. California and Florida accounted for 30% of cases in the study. Of the six states with the highest number of Alzheimer's disease cases (California, Florida, New York, Pennsylvania, Illinois, Texas) [15], all but Texas (ranked 10^th^) were in the top 6 most frequent states. Virginia ranked fourth, possibly representing the extensive search and rescue efforts of that state [4]. These figures are consistent with previous research with a higher proportion of males, large age span, and proportional representation by state [7].

**Table 1:** Demographic characteristics of sample

	Total Cases (**% by column**)	Found Alive (**% by row**)	Found Dead (**% by row**)
Incidence	325	222 (68)	103 (32)

Male	206 (63)	138 (67)	68 (33)

Most prevalent states	California (16) Florida (14)	California (18) Florida (14)	Florida (15) California (14)

Types of residence	298	205	93

Home	222 (74)	147 (66)	75 (34)

Nursing home	17 (6)	14 (82)	3 (18)

Assisted living facility	18(6)	10 (55)	8 (45)

Domiciliary facility	9(3)	7(78)	2(22)

Others	32 (11)	27 (84)	5 (16)

Mean age (range)	76 (40-95)	76 (40-93)	77 (49-95)

Approximately 70% of PWD live in the home setting reflecting the 75% of our sample that was residing at home at the time of a missing incident. Although greater numbers live in nursing homes than domiciliary care facilities (assisted living and board-and-care homes), a greater percentage of missing incidents occurred from the domiciliary facilities. This confirms the finding of our previous study with a proportionally higher percentage of individuals having missing incidents from domiciliary facilities. Other places last seen included the temporary residences of hospitals and out-of-town with family.

Of those who were walking, 53% were found within 1 mile and 75% within 5 miles. Only 11% of those who drove away were found within 5 miles of the PLS. For those who drove away, the range was 0.03 - 1745 miles with a median of 41.3.

As compared to our previous study [13], there were relatively more individuals missing while driving and relatively fewer missing after leaving the home when agitated, when outside the home with the caregiver or when the caregiver was distracted inside the home. This is likely due to using newspaper articles rather than the SafeReturn database to gather the cases with only the more remarkable cases reported in the newspaper.

Of those found alive, 72% were found by the next day while only 40% of those dead were found by the next day. Of those not found by the next day (n = 118), 51% were found alive and 49% were found dead. For those not found until at least the 5^th ^day after missing (n = 45), only 20% were found alive.

### Aim 1. Antecedents and Characteristics of Missing Incidents

The categories proposed in a previous study [5] fit the antecedent causes in this study population well and were used as the classification scheme of the antecedent event (see Table 2). The majority of individuals (58%) were engaged in an appropriate and permitted unsupervised activity just prior to the missing incident. Only examining those individuals who resided at home (n = 222) where independent activities are more likely to occur, this percentage increased to 72% indicating that the most predominant antecedent to a missing incident in community-dwelling PWD was conducting a normal and expected activity that was intentionally unsupervised.

**Table 2:** Context of the missing event

	Total Cases (**% of column**)	Found Alive (**% of row**)	Found Dead (**% of row**)
**Planned independent activities in the community**			

Normal outing driving	74 (28)	54 (73)	20 (27)

Normal outing walking	52 (20)	39 (75)	13 (25)

**Planned independent activities in the home**			

Outside home with caregiver	19 (7)	13 (68)	6 (32)

Independent in home while caregiver elsewhere	12 (5)	5 (42)	7 (58)

**Left during lapse of supervision, unplanned event**			

Caregiving facility	45 (17)	33 (73)	12 (27)

Home alone	25 (9)	14 (56)	11 (44)

Caregiver asleep	23 (9)	11 (48)	12 (52)

Drove away unexpected	6 (2)	3 (50)	3 (50)

Transportation error	1 (< 1)	1 (100)	-

Other event	3 (1)	2 (67)	1 (33)

**Left while supervised**			

PWD agitated and left home	6 (2)	4 (67)	2 (33)

**Total cases**	266 (100)	179 (67)	87 (33)

The other major cause of missing incidents was a lapse in supervision, often intentional as when an informal caregiver sleeps and at other times unintentional such as unattended exit at a professional care setting. The primary antecedents for community-dwelling PWD in this category were being left home alone and leaving at night while the caregiver was asleep. There was not sufficient information to examine the antecedents for those living in a formal care setting,

For individuals who went missing while driving (74 cases), the majority (80%) did so while on a trip that was permitted by caregivers. Only 11% of those missing while driving drove off without permission during the day and 4% drove off during the night. Of these 15% unattended exit cases, interestingly, several took place away from the home when the PWD, who was to wait alone in the car while the caregiver ran an errand, drove away alone before the caregiver returned. The remaining 5% of driving cases constituted missing couples with at least one member being diagnosed with dementia.

For those who left during an unintentional lapse in supervision, it is less clear how to determine whether the event was unexpected and unpredictable. A small percentage of individuals (8%) indicated they were trying to get to a former residence or work location or a relative's home. The most common reason that caregivers provided was that the PWD just got lost. No stories indicated that the individual had been pacing, lapping or walking frequently in the PLS and as a result of that activity, the individual left a supervised area and was unable to return.

Lapse of supervision, whether planned or unplanned, was an antecedent characteristic in all cases except for 6 (2%) individuals. These 6 individuals left the home after becoming agitated and with the knowledge of the caregiver. However, these individuals were subsequently unsupervised after they left the home, and then went missing.

### Aim 2. Differences between those found alive and dead

Thirty percent of individuals were found dead. This number doesn't reflect a typical ratio of cases found alive versus dead since the number found dead in the general population is likely below 1% as most longitudinal studies haven't recorded any deaths [2,16]. The over-representation is a function of the sampling strategy of using newspaper articles, but it does provide an excellent sample to compare differences of those found alive versus dead.

It took significantly longer to find those who died (independent samples median test, *p *< .001). While 90% of those found alive were found within 2 days after being reported missing, only 50% of those who died were found in this time frame (see Table 3). The 90% recovery mark for the group that died was not reached until greater than 26 days after the PWD was reported missing.

**Table 3:** Total days to find - individuals found dead

# of days	Frequency	Cumulative percent	Died by exposure	Died by drowning	Hit by Vehicle
Same	12	12.4	3	3	3

1	27	40.2	12	10	3

2	9	49.5	4	2	0

3	8	57.7	3	2	0

4	5	62.9	3	0	0

5	3	66.0	2	0	0

6	2	68.0	0	0	0

7	3	71.1	2	0	0

8-14	13	85.6	6	2	0

15-30	4	89.7	20	18	0

> 30	10	100.0	2	3	0

**Total**	**97**		**57**	**37**	**6**

For those who went missing while walking, those found dead were significantly closer than those found alive (*X^2 ^*= 14.74, *p *= 0.01) with 50% of those found dead within 0.5 miles of the PLS (see Table 4). However there was no significant relationship between distance and disposition for those who were missing while driving (*X^2 ^*= 6.88, *p *= 0.33). The 24 drivers who were found dead succumbed to exposure; 7 of these were found inside the car, and the rest were found very close to the car (X = 0.6 miles, range = 0.01 - 2 miles).

**Table 4:** Total days to find - individuals found alive

# of days	Frequency	Cumulative percent
Same day	68	31.3

1	89	72.4

2	38	89.9

3	12	95.4

4	1	95.9

5	1	96.3

6	1	96.8

8	2	97.7

10	1	98.2

12	2	99.1

13	1	99.5

20	1	100

**Total**	**217**	

Examining which antecedents were associated with those found dead, the overall *X*^2 ^test was not significant (15.37, *p *= 0.12). However three situations had higher within group percentages of PWD found dead than alive. These situations, caregiver distracted in the home (58% found dead), caregiver asleep (54%) and left home alone (48%), all occurred at a time in which the individual was in the home and unsupervised. Thus in community-dwelling PWD those leaving from the home during a lapse of supervision versus during an independent community activity may be at higher risk of death.

Males were not more likely to die (*X^2 ^*= 1.96, *p *= 0.38) nor was age a significant factor (*t *= 1.35, *p *= 0.18). Overall type of residence was not associated with a greater risk of death (*X *^2 ^= 7.62, p = 0.18), however, 44% of those whose PLS was an assisted living facility were found dead confirming a previous finding of a high rate of death when missing from this care setting [6].

### Aim 3: Differences between missing incidents and wandering

When the characteristics of missing incidents are compared to the formal definition of *wandering *(locomotion behavior, repetitive, frequent, temporally- and spatially-disordered [3]p. 696), there are notable differences. The first characteristic of wandering, that of locomotion behavior characterized by a repeated activity, is not consistent with the phenomenon of missing incidents in PWD. It is key that 58% of the cases (72% of community-dwelling cases) occurred during the conduct of a usual everyday activity, not while walking in a predictable pattern in an area. Walking was only one of several methods of travel that were used during a missing event which also included driving, riding a bicycle or taking public transportation. Furthermore, the characteristics of the activity after leaving, either on foot or driving, do not fit the patterns of pacing, lapping or random. Some individuals secluded themselves in natural areas less than 0.5 miles of the PLS (26%) and remained there until found, demonstrating little locomotion activity. In contrast others walked greater than 5 miles away (maximum = 19.6 miles) or drove many miles away (maximum = 1745 miles).

Second, while wandering is frequent and repetitive, PWD generally do not have multiple instances missing events. In this sample, only two individuals had greater than one incident with each of those having 2 incidents. In the study of the SafeReturn database, only 6% of the sample had been involved in more than incident [5]. In a prospective study over a 5-year period, there were 43 subjects who had to be returned by someone else after leaving the home. Of these, 19 had a single incident and 18 had 2-5 incidents. Only 6 subjects had greater than 5 incidents in the 5-year period.

Wandering is characterized by both temporal- and spatial-disorientation. For over half the sample, the incident was not temporally-disordered as they were on a usual, expected activity occuring at an appropriate time of day. Only one group clearly had temporally-disordered activity - the 21 subjects who left their residence while the relative caregiver was asleep. Many of these left clothed inappropriately (pajamas, undergarments or naked) or without proper clothing for cold weather. Seven of the 21 were not noticed to be missing until the caregiver awoke in the morning.

Spatial disorientation is likely a key feature of missing incidents. Since 58% of the incidents occurred in the course of conducting a usual and expected activity, some trigger must have occurred to alter the conduct of that activity. Since several studies suggest a link between spatial disorientation and a missing incident [2,14,17], it may be that the PWD becomes spatially disoriented during their usual activity and are unable to recover from way finding errors that may occur.

## Discussion

Most importantly, the findings of this study continue to identify distinct and important differences between *wandering*, as conceptually defined, and a missing incident. Confusion between the terms may result in the caregiver incorrectly assessing the risk of a missing incident and not taking appropriate action to possibly prevent that from occurring. Additionally, few caregivers take advantage of programs that will facilitate a safe return if the individual is found by strangers and can't state essential information [18]. In the United States, the most common program to provide this is the MedicAlert SafeReturn™ program and in Canada a similar program is Safely Home.

In discussions with caregivers on actions to take in order to prevent a missing incident, it is critical to stress that most events occurred when the PWD was engaged in a normal, independent activity. It is important to identify specific strategies that can be used in during usual, independent activities, as many caregiver-reported strategies such as "keeping a closer eye'" do not fit the antecedents and circumstances of a missing incident.

Since these events are unexpected and unpredictable, it is critical to ensure that all PWD have a means of identification with them at all times. Besides the national efforts above, a number of communities have also initiated local alert systems in which a database is kept in case of an incident [19]. Even a bracelet with essential information would be helpful Because PWD are often unable to state their name, address or phone number when found, these identification systems facilitate an easier return once the individual has been located.

Next, locating and tracking interventions can be considered. These would be most important for those at high risk of a missing incident, for instance males with high levels of independent activity in the community. Locating technologies, such as radiofrequency technologies that can find an individual in buildings, under tree cover, etc., are engaged once the individual is determined to be missing. Radio frequency identification locator systems are an example of this technology and can be ordered through local law enforcement agencies who have obtained the equipment. Tracking strategies use a combination of satellite and cellular signals to track the general location of an individual at any given time, similar to using a GPS system in a car. These systems do not work well when the individual has cover overhead such as trees or bridges, or in buildings, since the signal cannot be transmitted. Additionally, the reported location can be many yards from the actual location of the individual. Thus locating technologies would be the preferred technology if the goal was to find a lost PWD.

Traditional strategies recommended for 'wandering' in its broadest sense will only impact a portion of the antecedents to this event. These would include actions such as techniques to prevent exit from a door (changing lock type, disguising the door) and methods to identify exits from doors (exit door alarms). Additional strategies will need to be developed to address the main antecedents to a missing incident.

An on-going evaluation of the individual's way finding skills is critical. It appears that a way finding error is a critical linchpin to a missing incident. Since almost all individuals are on a customary activity or in a familiar location as the antecedent of the event, something distinct occurs to cause them to become lost. Likely, this is a way finding error that may be associated with visual or spatial disorientation. This critical way finding error cascades into a series of problems potentiated by other dementia deficits, such as lack of judgment, impaired abstract thinking, and impaired memory. These combine to make it impossible to recover from the way finding error. The Trail Making Test B, a commonly used neuropsychological test, provides information on visual search, scanning, mental processing speed and flexibility, and executive brain function. The test is sensitive to neurological impairments and can be used to assess way finding ability in persons with dementia [20].

A high number of cases we found occurred while driving. A useful strategy would be the implementation of driving assessment and retirement strategies with all persons who have been diagnosed with dementia, particularly those scoring poorly in way finding tasks [21]. The education about and provision of alternative forms of transportation are critical to the success of driving retirement.

The primary limitation of this study is that the data were derived from cases that required both a call to law enforcement to assist in finding the individual and a report in a newspaper. Likely these cases are the minority of the total number of missing incidents in PWD with caregivers finding the majority of individual without law enforcement help [2]. Thus while the statistics reported are relevant for the cases who require law enforcement assistance, they are not reflective of the entire population of missing incidents in which there would be a much lower mortality rate and more rapid recovery times.

## Conclusions

This research supports the mounting evidence that the concept of *wandering*, in its formal sense, and *missing incidents *are two distinct concepts. It will be important to further develop each concept identifying assessment parameters that can be used by caregivers to accurately understand each of these problems that occur in PWD. This will allow a more targeted intervention strategy and hopefully a reduction in negative outcomes.

## Competing interests

The authors declare that they have no competing interests.

## Authors' contributions

MR designed and supervised the collection and analysis of data. MR and CG were responsible for the majority of manuscript writing. SV, CL, RF, NM, and HA were responsible for finding, retrieving and abstracting data from the newspaper articles. SV and CL also built and cleaned the dataset used for analysis. CG was responsible for additional data checking, completing text analysis for some of the variables and completing the tables for the document. All authors read and approved the final manuscript.

## Acknowledgements

Publication of this article was funded in part by the University of Florida Open-Access Publishing Fund.

## Pre-publication history

The pre-publication history for this paper can be accessed here:

http://www.biomedcentral.com/1471-2318/11/28/prepub

## References

[B1] Carr D Muschert GW Kinney J Robbins E Petonito G Manning L Brown JS Silver alerts and the problem of missing adults with dementia Gerontologist 50 2 149 157 10.1093/geront/gnp102 19556393

[B2] McShane R Gedling K Keene J Fairburn C Jacoby R Hope T Getting lost in dementia: a longitudinal study of a behavioral symptom Int Psychogeriatr 1998 10 3 253 260 10.1017/S1041610298005365 9785146

[B3] Algase DL Moore DH Vandeweerd C Gavin-Dreschnack DJ Mapping the maze of terms and definitions in dementia-related wandering Aging Ment Health 2007 11 6 686 698 10.1080/13607860701366434 18074256

[B4] Koester RJ The lost Alzheimer's and related disorders subject: New research and perspectives Response 98 NASAR: 1998; Chantilly, VA 1998 165 181

[B5] Rowe M Glover J Antecendents, descriptions and consequences of wandering in cognitively-impaired adults and the Safe Return (SR) program American Journal of Alzheimer's Disease and Other Dementias 2001 16 6 344 352 10.1177/153331750101600610 11765859 PMC10833874

[B6] Rowe MA Bennett V A look at deaths occurring in persons with dementia lost in the community Am J Alzheimers Dis Other Demen 2003 18 6 343 348 10.1177/153331750301800612 14682082 PMC10833970

[B7] Rowe MA Feinglass NG Wiss ME Persons with dementia who become lost in the community: a case study, current research, and recommendations Mayo Clin Proc 2004 79 11 1417 1422 10.4065/79.11.1417 15544021

[B8] Flaherty G Tobin TS Silverstein NM Dementia and wandering behavior: Concern for the lost elder 2002 New York: Springer Pub. Co.

[B9] Butler JP Barnett CA Window of wandering Geriatr Nurs 1991 12 5 226 227 10.1016/S0197-4572(06)80008-X 1667165

[B10] Aud MA Dangerous wandering: elopements of older adults with dementia from long-term care facilities Am J Alzheimers Dis Other Demen 2004 19 6 361 368 10.1177/153331750401900602 15633945 PMC10833955

[B11] Moore DH Algase DL Powell-Cope G Applegarth S Beattie ER A framework for managing wandering and preventing elopement Am J Alzheimers Dis Other Demen 2009 24 3 208 219 10.1177/1533317509332625 19357378 PMC10846189

[B12] Hunt LA Brown AE Gilman IP Drivers with dementia and outcomes of becoming lost while driving Am J Occup Ther 64 2 225 232 10.5014/ajot.64.2.225 20437909

[B13] Rowe MA Glover JC Antecedents, descriptions and consequences of wandering in cognitively-impaired adults and the Safe Return (SR) program Am J Alzheimers Dis Other Demen 2001 16 6 344 352 10.1177/153331750101600610 11765859 PMC10833874

[B14] Tu MC Pai MC Getting lost for the first time in patients with Alzheimer's disease Int Psychogeriatr 2006 18 3 567 570 10.1017/S1041610206224025 16867206

[B15] Association As 2009 Alzheimer's disease facts and figures Alzheimer's & Dementia 2009 5 3 19426951 10.1016/j.jalz.2009.03.001

[B16] Rowe MA Ahn H Benito AP Stone H Wilson A Kairalla J Injuries and unattended home exits in persons with dementia: A 12-month prospective study Am J Alzheimers Dis Other Demen 2010 25 27 31 10.1177/1533317508323138 19001350 PMC2821464

[B17] Pai MC Jacobs WJ Topographical disorientation in community-residing patients with Alzheimer's disease Int J Geriatr Psychiatry 2004 19 3 250 255 10.1002/gps.1081 15027040

[B18] Bass E Rowe MA Moreno M McKenzie B Expanding participation in Alzheimer's association Safe Return by improving enrollment Am J Alzheimers Dis Other Demen 2008 23 5 447 450 10.1177/1533317508320974 18632877 PMC10846000

[B19] Cook M Law enforcement builds database to help people with special needs Californian 2010 San Diego

[B20] Ashendorf L Jefferson AL O'Connor MK Chaisson C Green RC Stern RA Trail Making Test errors in normal aging, mild cognitive impairment, and dementia Arch Clin Neuropsychol 2008 23 2 129 137 18178372 10.1016/j.acn.2007.11.005 PMC2693196

[B21] Eby DW Molnar LJ Driving fitness and cognitive impairment issues for physicians JAMA 2010 303 16 1642 1643 10.1001/jama.2010.495 20424255

